# A mixed-methods study exploring adherence to the referral of severely sick children in primary health care in Southern Ethiopia

**DOI:** 10.1186/s13690-021-00681-6

**Published:** 2021-09-02

**Authors:** Habtamu Beyene, Dejene Hailu, Henok Tadele, Lars Åke Persson, Della Berhanu

**Affiliations:** 1 Department of Maternal, Newborn, and Child Health, and Nutrition, Southern Nations, Nationalities & Peoples Regional Health Bureau, Hawassa, Ethiopia; 2 College of Medicine and Health Sciences, School of Public Health, Hawassa University, Sidama, Hawassa, Ethiopia; 3 College of Health Sciences, Department of Paediatrics and Child Health, Addis Ababa University, Addis Ababa, Ethiopia; 4 Faculty of Infectious and Tropical Diseases, London School of Hygiene and Tropical Medicine, London, UK; 5 Health System and Reproductive Health Research Directorate, Ethiopian Public Health Institute, 5654 Addis Ababa, Ethiopia

**Keywords:** Adherence to referral guideline, Childhood referral, Referral compliance, Severe illnesses, Sick child

## Abstract

**Background:**

We have shown that Ethiopian primary healthcare providers refer only half of the severely sick children who, according to guidelines, should get an urgent referral. Frequently parents of referred ill children don’t bring their children to the next level. We aimed to describe the referral of severely ill Ethiopian children based on primary healthcare register reviews and explore health care providers’ and parents’ perceptions regarding factors that hinder or enhance referral.

**Methods:**

A mixed-methods study was conducted in 11 districts and a town administration of the Hadiya zone in Ethiopia’s Southern region from May to June 2019. Data collection included interviews and focus group discussions with healthcare providers, key informant interviews with parents of sick children who had been referred, and reviewing registers of sick children treated during the last 12 months at health posts and health centres. We analysed the association between healthcare providers’ and sick children’s characteristics and providers’ compliance with referral guidelines for sick children 0–59 months old. Content analysis was undertaken to explore the perceived factors that influenced referral and adherence to referral from providers’ and parents’ perspectives.

**Results:**

Healthcare providers did not refer nearly half of the severely ill children that should have been referred, according to guidelines. Providers who had received in-service training on child healthcare were more likely to adhere to referral guidelines. The severity of the child’s illness and mobile phone communication and transport availability were perceived to be positively associated with adherence to referral guidelines. Lack of knowledge of treatment guidelines and skills, and high health worker workload, were among the factors perceived to be linked to lower adherence to guidelines. The healthcare providers considered parents of referred sick children as having low compliance with the referral advice. In contrast, parents had the opinion that compliance with a referral for sick children was high. Perceived awareness of severity of the child’s illness, ability to afford referral costs, and availability of transport or ambulance services were perceived to motivate parents to take their children to the referral facility. Traditional illness perceptions, lack of confidence in the referral site’s medical care, and a long distance were perceived to hurdle caregivers’ referral compliance.

**Conclusions:**

We found that the healthcare providers’ adherence to referral guidelines was not optimal. Care providers and parents had divergent opinions on parents’ compliance with referral advice. Factors related to the health system, family economy, and available ambulance services influence whether care providers and parents pursued severely ill children’s referral. Adequate referral of sick children is an aspect of primary healthcare quality that is essential to avoid unnecessary under-five deaths.

**Supplementary Information:**

The online version contains supplementary material available at 10.1186/s13690-021-00681-6.

## Background

More than half of the global 5.3 million under-five deaths occurred in Sub-Saharan Africa in 2018. Infectious diseases remained the leading causes of death [1].

Despite a remarkable improvement during the Millennium Development Goal era [2], Ethiopia still has a relatively high under-five and neonatal mortality at 55 and 30 deaths per 1,000 live births, respectively [3]. Lower respiratory tract infections, diarrhoeal diseases, and neonatal causes (mainly preterm birth complications, asphyxia, and neonatal sepsis) were the leading causes of death [4].

The integrated management of newborn and childhood Illnesses (IMNCI), the integrated case management of childhood illnesses (iCCM) and the community based newborn care (CBNC) programmes were among the strategies implemented in Ethiopia to reduce under-five deaths [5–7]. Referral of children with severe illnesses is a critical element in these programmes.

At first- and referral-level facilities, adherence to referral guidelines by healthcare providers and users is essential [8]. However, studies in Ethiopia and Malawi showed that only around half of the severely sick children, who should get urgent referral, were sent to the next health system level [9–11].

Different studies in low-income countries have revealed that frequently referred patients do not reach the receiving health facilities [12–14]. High referral compliance is an important indicator of an effective referral system. It involves multiple steps; from the initial recognition of a disease that needs access to specific services to the successful management of the illness at the higher-level facility [15].

Parents’ acceptance that the child needs referral and their compliance with this recommendation is influenced by various factors [8]. The perceived need of a referral (disease severity), the experiences or perceptions of the referral facility (quality) and the cost (time and resources) may determine referral compliance [8, 16].

A South African study revealed that caregivers’ compliance with the primary-level referral was less than half [17]. Younger caregivers were more likely to comply. In a Ugandan study, referral completion was higher among children with danger signs [18]. However, when children received pre-referral treatment, they were less likely to complete the transfer to the next level.

The most commonly reported reasons for non-compliance with referral include transportation problems, family-related reasons (e.g., mother ill, nobody caring for other children, nobody accompanying the sick child, no permission to go), and perceived poor quality of care at the referral facility (e.g., unskilled staff, no medicines) [18–20].

In Ethiopia, research is scarce on healthcare providers’ adherence to referral guidelines and parents’ compliance with the referral advice they received for their severely ill children.

Hence, this study aimed to explore factors associated with primary healthcare providers’ adherence to referral guidelines for severely ill children and parents’ compliance with the referral advice.

## Methods

### Study design and setting

This study employed a mixed-methods approach, including a facility-based cross-sectional quantitative study and a phenomenological qualitative study. This mixed-methods design was used to obtain more comprehensive and convincing evidences [21]. For the quantitative data, the Ethiopian community-based newborn care, the integrated community case management, and the integrated management of newborn and childhood illnesses guidelines [22, 23] were used as the gold standard to evaluate whether the healthcare providers at the primary health care level adhered to the referral guidelines. These guidelines were used to examine whether the severely ill children were appropriately referred or not (Supplementary Tables 1 and 2).

The qualitative data collection allowed us to further explore and identify reasons linked with referral adherence. The qualitative data was also used to triangulate findings with different participants and also with the quantitative data. The qualitative study also investigated the perceived enablers and barriers to the caregivers’ compliance with referral advice. In a previous study, we found similar referral practices across a large number of districts in the four largest Ethiopian regions [24]. The present study is limited to one zone in the Southern Nations, Nationalities, and Peoples region, but may anyhow have sick child referral practices that resemble other Ethiopian regions.

The study was conducted in Hadiya zone of the Southern region of Ethiopia. The survey and qualitative study were conducted from May to July, 2019. The study area had a total population of nearly 1.7 million, and an under-five population of 263,675 at the time of the survey. There were 377 health posts and 62 health centres providing child health services [25]. The quantitative study covered all 11 districts and one town administration. The qualitative study was performed in two districts of the zone, namely Soro and Mirab Soro.

### Study population

The study units for the quantitative component of this study were selected health care providers who treated sick under-five children in the sampled primary health care units of the study area. In addition, records were reviewed of all under-five children with severe childhood illnesses during the last 12 months before the study. Information about referral was collected and compared with the relevant referral guidelines.

For the qualitative component, caregivers of under-five children with referral histories in the last 6 months before data collection were included. They shared their lived rich experiences and perceptions of factors that facilitate or hinder compliance with referral advice. Two health care providers serving at child health departments in the sampled health facilities were also included in the qualitative sub-study.

### Sample size

The sample size of the quantitative component was determined using a single population proportion formula with the following assumptions: a significance level of 5 %, 95 % confidence interval level, 50 % proportion of under-five children with severe illnesses who were actually referred, a design effect size of 2, and a non-response rate of 10 %. Thus, records of a total of 845 under-five children with severe disease classification were reviewed. In addition, the health facilities along with their health care providers were surveyed.

### Sampling procedure

A multistage sampling technique was used. A total of 377 health posts and 62 health centres providing child health services in Hadiya zone in 2018-19 were included. Based on a list of these primary healthcare facilities in all 12 districts of the zone, it was possible to select a random sample of health centres and health posts to represent the study districts. The samples were proportionally allocated to the districts and health facilities, using a multistage sampling.

The expected number of sick child consultations in Hadiya zone in the year 2018 -19 was estimated to be 131,838. Similarly, the expected number of sick young children requiring referral was estimated to be 13,184, considering that 10 % of the consulted under-five children required referral to higher levels [26]. The sample was randomly selected proportional to the under-five patient flow; 43 health posts providing the integrated community case management and the community-based newborn care, and 20 health centres providing the integrated management of newborn and child illnesses. The record review of sick children was performed until the required sample size from each facility was secured.

For the qualitative component, a purposive criterion-based sampling was employed. Accordingly, two districts with high under-five patient flow were selected. After that, a sample of two health centres and four health posts from each district was selected. Hence, for in-depth interviews, a total of 24 parents (two from each health facility), a total of 12 per each district, whose children had been referred during the last 6 months, were selected from the child healthcare registers. The parents were traced in their respective communities with the assistance from local women’s development group leaders and health extension workers.

Similarly, 24 healthcare providers (12 from health centres and 12 from health posts) who provided care to under-five children were selected from the two districts for focus group discussions (FGD) (a total of four FGDs).

The final sample size in the qualitative study was based upon the principle of saturation [27, 28].

### Data collection

We collected the quantitative and qualitative data at the same time and integrated our findings based on our study aims [21]. For the quantitative component, the record review of under-five children from treatment registers was employed to analyse whether children with severe illnesses or dangers signs were referred or not according to guidelines. The records of the sampled sick child consultations in the previous 12 months were reviewed. Information was obtained on children’s background, residence, signs and symptoms, disease classification or diagnosis, and referral status.

We interviewed healthcare providers using a pre-tested structured questionnaire. In order to complement the findings from the healthcare providers’ interviews, we performed two FGDs to explore the perceived factors that enable or hinder adherence to the existing referral guidelines.

In-depth interviews were performed with 18 parents using semi-structured interview guides to explore the lived experiences of referral and compliance with the referral advice. In addition, two additional FGDs were performed with healthcare providers to explore their perceptions of parents’ referral compliance. The interviews and FGDs were continued until data saturation was achieved.

With permission from the participants, the in-depth interviews and the FGDs were audio-recorded. Time and place of interviews were selected to be convenient for the study participants. The in-depth interviews were performed in the local language, Hadiya. In the FGDs, both Amharic and Hadiya languages were used. Local translators translated the Hadiya records into Amharic. Finally, the Amharic translation was transcribed into English by the first author.

Data were collected by experienced data collectors who were at least a college graduates and trained in the national iCCM/CBNC and IMNCI guidelines. Data collectors received a five-day intensive training on study procedures, questionnaires, data collection techniques, clinical guidelines, quality-assurance procedures, and study ethics. The data collection tools were pre-tested on the final day of the training. The first author supervised the data collection process.

### Data analyses

All questionnaire data were cleaned, checked for completeness and coded before entry. Descriptive statistics included frequency distributions, and proportions. Chi-square tests were employed to identify factors associated with healthcare providers’ adherence to the existing referral guidelines. Level of significance was set at *p*-value less than 0.05 using 95 % confidence level. Statistical analyses were done in Stata version 15.1 (Stata Corp LP, College Station, Texas, USA).

For the qualitative part, data were manually analysed using a qualitative content analysis according to Granheime and Lundman [29]. This process included open coding, abstracting, and creating classes. All individual interviews and FGDs were transcribed and the texts were read several times. Initial codes were determined and similar codes were allocated to the same category or theme. The categories or themes were grouped to form larger themes. The data analysis took differences and similarities between informants into consideration.

With the mixed-methods approach, the results of the quantitative and qualitative components were compared, synthesized and discussed.

## Results

### Characteristics of informants

Among the healthcare providers who participated in the quantitative study, 81 % were women, 54 % were below the age of 30 and a third had served less than 5 years. Data were collected from 43 health extension workers and 20 nurses providing child healthcare in 11 districts across Hadiya zone. In addition, data on 110 severely sick 0–59 days young infants and 733 severely sick children aged 2–59 months were abstracted from treatment registers of the 43 health posts and 20 health centres. The study also included the intended 18 key informant interviews with caregivers and four focus group discussions with healthcare providers. Among the caregivers included in the qualitative study, the majority were above the age of 30 and were more frequently parents of girls.

### Health facilities

A majority of the health posts offered community-based newborn care (CBNC) and integrated community case management (ICCM) services. All health centres offered integrated management of newborn and childhood illnesses (IMNCI) services. Nearly a fifth of the health posts and health centres had no treatment guidelines required to have for the management of sick 0–59 days and 2–59 months old children. More than half of the health facilities did not have any observed records of quality assurance activities for child health services. Nearly half of the health facilities did not have any referral transportation for sick children (Table 1).

**Table 1:** Characteristics of health facilities and care providers in Hadiya zone, Ethiopia (May – July 2019)

Characteristics	n	%
Type of health facility		
Health post	43	68
Health centre	20	32
Health facilities offering child health services		
Community-based newborn care at health posts (*N* = 43)	35	81
Integrated community case management at health posts (*N* = 43)	31	72
Integrated management of newborn and childhood illnesses (*N* = 20)	20	100
Availability of treatment guidelines of		
Community-based newborn care	35	81
Integrated community case management	36	84
Integrated management of newborn and childhood illnesses	16	80
Profession of health care providers working in child health department		
Health extension worker	43	68
Nurse	18	29
Health officer	2	3
Age of health care provider in years		
19–29	34	54
=> 30	29	46
Sex of health care provider		
Man	12	19
Woman	51	81
Service period of health care providers (years)		
< = 5	22	35
6–10	17	27
=> 11	24	38
Healthcare providers received in-service training on care and treatment of		
0–59 days sick young infants	38	60
2–59 months sick children	38	60
Healthcare providers received supportive supervision on care and treatment of		
0–59 days sick young infants	14	22
2–59 months sick children	16	37
Healthcare providers perceived regular adherence to the treatment guidelines of		
0–59 days sick young infants	42	67
2–59 months sick children	32	74
Availability of transportation facility for referral of sick children		
No	27	43
Yes	36	57
Health providers made advance call to alert the next level of care when referral of the last child was done?		
No	27	43
Yes	36	57
Experience of back referral by health facilities		
No	52	82
Yes	11	18
Healthcare providers’ perception of parents’ compliance with referral advice		
Not complying	37	59
Yes, complying	26	41
Facilities have observed records of quality assurance activity		
No	37	59
Yes	26	41
Number of 0–59 days sick young infants		
Who consulted the health facilities	1872	
With possible severe bacterial infections	110	6
With possible severe bacterial infections referred	66	60
Number of 2–59 months sick children		
Who consulted the health facilities	22,037	
With severe diseases that require referral	733	3
With severe diseases who were referred	397	54

### Healthcare providers

A significant proportion of the healthcare providers had not received in-service training on sick child treatment guidelines (Table 1). Only a few of these providers had got on-the-job supportive supervision from a higher health system level. Only two-thirds of the healthcare providers perceived that they adhered to the existing sick young infant guidelines and three-quarters to the sick child guidelines. Nearly half of the healthcare providers claimed that they made an advance call to alert the next level of care when they had referred a sick child last time. More than half of the surveyed healthcare providers perceived that caregivers often did not comply with the referral advice.

### Referral rate and caregivers’ referral compliance

Out of the 1872 sick 0–59 days sick young infants who consulted the facilities for their illnesses, 110 (6 %) had possible severe bacterial infections that required referral. Of these, 60 % were referred to the next level of care. Similarly, 733 (3 %) of the older children who consulted the health facilities had severe diseases that required referral and 54 % of these were appropriately referred to the next level of care.

More than half of the caregivers of the referred children were, according to the healthcare providers, less likely to adhere to the referral advice (Table 1). However, the qualitative key informant interview showed that the majority of parents claimed that they had taken their referred children to the referral health facility.

### Adherence to referral guidelines

There were no differences in adherence to the referral guidelines across the different professions, age groups, gender, periods of service, or between facilities with and without ambulances (Table 2). There were no any differences in appropriate referral between health facilities with records of quality control activity and those without (Table 2).

**Table 2:** Health facility and child factors associated with adherence by healthcare providers to referral guidelines for 0 – 59 months old sick children in Hadiya zone of Ethiopia

Characteristics	Healthcare providers’ adherence to referral guideline for sick 0 – 59 days sick young infants			Healthcare providers’ adherence to referral guideline for sick 2 – 59 months sick children		
	Appropriate decision *N* = 66 n (%)	Inappropriate decision *N* = 44 n (%)	*P*-value	Appropriate decision *N* = 397 n (%)	Inappropriate decision *N* = 336 n (%)	*P*-value
Profession of health care providers working in child health department						
Health extension worker	38 (58)	28 (42)	0.690	218 (55)	180 (45)	0.890
Nurse	28 (63)	16 (37)		179 (53)	156 (47)	
Age of healthcare provider in years						
19 – 29	35 (55)	29 (45)	0.308	195 (49)	207 (51)	0.179
=> 30	31 (67)	15 (33)		202 (61)	129 (39)	
Sex of healthcare provider						
Male	50 (61)	32 (39)	0.766	265 (53)	231 (47)	0.675
Female	16 (57)	12 (43)		128 (58)	93 (42)	
Service years of healthcare providers in years						
< = 5	21 (53)	19 (47)	0.561	144 (49)	149 (51)	0.151
6 – 10	22 (61)	14 (39)		86 (48)	95 (52)	
=> 11	23 (68)	11 (32)		167 (64)	92 (36)	
Availability of ambulance or other transport for referral of sick children						
No	28 (62)	17 (38)	0.710	128 (56)	101 (44)	0.836
Yes	38 (58)	27 (42)		269 (53)	235 (47)	
Facilities have records of quality assurance activity						
No	40 (61)	26 (39)	0.910	122 (43)	161 (57)	0.089
Yes	26 (59)	18 (41)		275 (61)	175 (39)	
Healthcare providers received in-service training on child care and treatment						
No	30 (55)	25 (45)	0.217	152 (46)	180 (54)	0.015
Yes	36 (65)	19 (35)		245 (61)	156 (39)	
Healthcare providers received supportive supervision on child care and treatment						
No	52 (59)	36 (41)	0.797	331 (55)	272 (45)	0.701
Yes	14 (64)	8 (36)		66 (51)	63 (49)	
Age of sick child						
1^st^ week	13 (57)	10 (43)	0.605			
2 – 4 weeks	22 (55)	18 (45)				
5 – 8 weeks	31 (66)	16 (34)				
2 – 11 months				114 (54)	97 (46)	0.985
12 – 23 months				114 (55)	95 (45)	
24 – 59 months				169 (54)	144 (46)	
Sex of sick child						
Boy	38 (61)	24 (39)	0.776	218 (55)	181 (45)	0.762
Girl	28 (58)	20 (42)		179 (54)	155 (44)	
Caregivers’ compliance with referral advice as perceived by healthcare providers						
Low	28 (61)	18 (39)	0.889	152 (56)	158 4)	0.661
High	38 (59)	26 (41)		245 (53)	218 (47)	

The adherence to referral guidelines of the older sick children was higher when healthcare providers had received in-service training (*p* = 0.015), Table 2.

In the focus group discussions, the severity of the child’s illness, availability of healthcare providers who were trained on the CBNC, ICCM and IMNCI guidelines, availability of ambulance services, and assignment of a focal person for managing sick children were factors mentioned to enhance adherence to the referral guidelines.

The healthcare providers also suggested that lack of knowledge and skills were barriers to follow the treatment guidelines.

*“I feel that some of the referred cases could have been managed by ourselves at the health centre level. However, we sometimes rush to refer the sick child to hospital as we feel that we lack the knowledge and skills since we are not trained in some child health programs such as community-based newborn care, integrated management of newborn and childhood illnesses and severe acute malnutrition programs. Some of us are working with the knowledge and skills we acquired while we were in college, without any further update” (Clinical nurse, 24 years, focus group discussion).*

The healthcare providers also mentioned that a high workload, lack of ambulances, scarcity of essential medicines and diagnostics, but also carelessness of some health workers could lead to inappropriate referral.

*“While I was working at the rural health centre, I remember that there were times the health professionals of the health centre took risks to treat the severely sick children that deserved referral care only for the caregivers’ economic situation. We were taking the risk only to prevent them from returning back to home, without getting any cure for their sick children”* (Clinical nurse, 28 years, focus group discussion).

### Factors associated with parents’ referral compliance

More than half of the parents of the referred children were perceived by the healthcare providers to have a low compliance with referral advices. However, in the interviews, the vast majority of the parents claimed that they had taken their referred children to the referral health facility. These interviewees stressed the importance of complying with a referral advice.

The FGDs of the healthcare providers and the in-depth interviews with parents of the referred children showed that caregivers were encouraged to comply with referral advice when they had the ability to afford referral costs and were members of a community-based health insurance scheme (CBHI). CBHI is a scheme in which households are required to enrol to protect them against catastrophic out-of-pocket healthcare expenditure. The availability of ambulances, a good-quality health extension program, and a perceived availability of better care at the referral site were thought to encourage parents to comply with a referral advice.

There were factors that hinder parents from taking their referred children to the referral facility. Healthcare providers mentioned poverty; families that could not afford referral costs.

“*Because of financial problems, families of sick children sometimes do not accept the referral and prefer to take their severely sick children back home or to the traditional healer“(*Health extension worker, 28 years, focus group discussion).

They could also object to the referral advice due to traditional illness perceptions and practices. Also, a long distance to the referral site could demotivate caregivers from complying with referral advice.

*“Severely sick children who were coming from rural settings were more affected by the absence of ambulance services”* (Clinical nurse, 28 years, focus group discussion).

Unavailability of transportation, lack of confidence in medical care at the referral site, and perceived disrespectful care were perceived as hurdles to parents’ referral compliance.

*“Nowadays, more emphasis has been given to the pregnant or labouring mothers. The children are getting less attention. Hence, this situation should be corrected. Although there are ambulance services in our area, they are only serving pregnant and labouring mothers. No services for severely sick children and other critical conditions.” (A health extension worker, 38 years, focus group discussion).*

In the in-depth interviews with mothers, lack of money was mentioned to be the most prominent reason for non-compliance with referral advice. Other reasons mentioned were lack of hope of improvement of the severely sick child, and that nobody took care of the children remaining at home.

An association of severe illnesses with perceptions like the evil eye was also mentioned to be a barrier for referral compliance.

A mother of a 3-years-old child, who had been referred for a severe illness, stated:

*“I was told by my neighbours not to take my sick child to the health post and that my daughter’s problem was the evil eye. Some people in our community believe that severely sick children with such kind of illnesses (cough and high fever) will die if they are given an injectable medication. I took my daughter to the referral site despite all this.”* (Mother, 30 years, key informant interview).

## Discussion

We found that over half of the healthcare providers complied with referral guidelines when managing 0–59 months old children with severe illnesses. Healthcare providers who had received in-service training on child treatment were more likely to appropriately refer severely sick older children according to guidelines. Some factors were perceived to be positively associated with referral adherence according to guidelines: the severity of the child’s illness, availability of a healthcare provider who had been trained on treatment guidelines, and availability of referral logistics. Other factors were considered to be linked to lower adherence to guidelines: high workload, lack of ambulances, stock-out of essential medicines and diagnostics. Parents of referred severely sick children were more ready to accept the referral advice if they recognised the severity of the child’s illness. Compliance was also related to ability to afford cost of transportation, medicines and care at the referral site. Being a member of a community-based health insurance scheme could also influence compliance. Availability of transport or ambulance services and a strong health extension program were also mentioned to motivate parents’ referral compliance. Families could also object to a referral advice due to traditional illness perceptions and practices. A lack of confidence in the medical care at the referral site and a long distance could be a hurdle to comply with referral.

We found that the healthcare providers did not refer more than a quarter of sick young infants and older children with severe illnesses that require referral. In a previous study in four Ethiopian regions, we found that nearly half of the severely sick young infants were not referred [24]. Poor adherence to guidelines could result in misuse of antibiotics, missing treatment, and increased lethality in severe cases [30]. Hence, actions to improve adherence to referral guidelines are needed as part of improved quality-of-care.

Several reasons were stated for the lack of adherence of the healthcare providers to referral guidelines. At the health worker level, despite knowing the need for referral they may act differently. In a study in Tanzania, only 25 % of children classified to be severely ill at the primary care level were referred. In that setting, the healthcare providers disagreed with the guidelines and considered management and treatment possible at the primary care unit [31]. Parents might also refuse referral in spite of recommendations from the health worker [13]. Mother’s illness [20], distance to the higher-level facility, travel cost, weather, and perceived poor quality of care at the referral facility have all been shown to limit referral acceptance by parents [18, 19], which in turn impacts the health providers’ adherence to guidelines.

We also found that the probability of adherence to referral guidelines of the older sick children was higher when healthcare providers had received in-service training. In a recent Ethiopian study and a systematic review, training of healthcare providers on national referral protocols and guidelines was recommended for an improved referral process [32, 33].

The qualitative study also indicated that the severity of the child’s illness prompted healthcare providers to adhere to referral guidelines. This finding was consistent with a previous Nigerian study [34]. Hence, this calls for strengthening the healthcare providers’ ability to identify severely sick children requiring urgent referral [19], which in turn could help the efforts being made to reduce the already set SDG targets of reducing newborn and under-five deaths [35].

The healthcare providers also suggested that lack of knowledge and skills were barriers to follow the referral guidelines. This finding might be linked to the insufficient knowledge of the health extension workers to accurately assess and classify childhood illnesses, as shown in a previous Ethiopian study [36]. Hence, healthcare providers need to be better trained in the care and treatment of newborns and under-five children in order to improve their performance of the referral system.

Although a reasonable number of ambulances were available in each district, we found that a lack of ambulances made available for sick children also hindered the healthcare providers to make appropriate referral decisions. This finding was similar to a Malawian study, where this problem was among the main challenges associated with implementation of the WHO guideline [37].

The scarcity of essential medicines and diagnostics were mentioned to force healthcare providers to inappropriately refer care-seeking children. This was also the case in a Tanzanian study, in which lack of essential drugs and supplies were among the main challenges in the implementation of the integrated management of childhood illnesses [38].

As expected, the qualitative study showed that parents as well as service providers perceived the severity of the child’s illness to motivate the caregivers to take their referred children to the referral facility. This finding was consistent with studies in Uganda [18] and Burkina Faso [15]. Hence, this calls for enhancing the awareness of the communities to identify children with signs and symptoms of severely sick children requiring urgent referral.

According to the healthcare providers, lack of willingness to accept and objection of the referral advice were among the barriers that were perceived to negatively affect caregivers’ referral compliance. Hence, effective counselling to the caregivers on the whole referral process, including the reason and importance, may counteract these problems [39].

Like mentioned in previous studies in Ethiopia [32], and India [40], longer distance to the referral site may hinder caregivers from taking their severely sick children to the referral site. Similarly, lack of money for transportation and health care costs was among the conditions that discouraged the caregivers from complying. This was also found in Burkina Faso [15], Uganda [18], and Bangladesh [41]. Hence, improving the coverage of enrolment in the existing community-based health insurance schemes may help to reduce this problem.

An Ethiopian health economics study indicated that an ambulance-based referral system was highly cost-effective for emergency obstetric and newborn care [42]. We found that ambulances were often not available for sick children but dedicated for pregnant and labouring mothers. The unavailability of ambulances for sick child referral was perceived to inhibit caregivers from taking their severely sick children to the referral site. Hence, measures need to be taken so that all critical and emergency cases including newborns and under-five children could benefit from ambulance services to avoid delays and avoidable deaths.

This study also found that perceived poor quality of care at the referral site could demotivate parents to take their children to the referral site. This finding was consistent with previous studies from Afghanistan [19], and Bangladeshi [43]. The quality of care for newborns and under-five children both at the referring and receiving health facilities need to be enhanced.

In this study we triangulated the quantitative findings with qualitative results to answer our research questions on referral compliance. It is probably the first Ethiopian study that assessed referral compliance using a mixed-methods approach. Though we have referral adherence information from the healthcare providers and caregivers’ compliance with a referral advice for severely sick children, we have not followed individual children and captured their information to evaluate compliance and outcome. Recall bias might have been a concern of this study. We tried to counter this problem by collecting some of the data from records, when applicable. As this study was performed in only one zone of the southern region of Ethiopia, the generalisability is limited. It should, however, be noted that we in an earlier study of sick child referral found limited variation across four Ethiopian regions [24]. Only a few associations between background characteristics and healthcare providers’ adherence to the referral guidelines were observed in the quantitative analyses. The relatively small sample size may have limited the possibility of displaying such associations.

## Conclusions

This study has shown that adherence of the healthcare providers to referral guidelines and compliance of parents with a referral advice were not optimal. Several family and health system related factors hampered the referral system. Healthcare providers’ adherence to care and treatment guidelines need to be enhanced by supportive supervision and clinical mentoring, and structural barriers to a well-functioning referral system should be removed. Community engagement may improve compliance with referral decisions. Further research is needed to identify strategies to improve the referral component in a well-functioning primary healthcare system.

## Supplementary Information

**Additional file 1: Supplementary table 1.** CBNC Classification table for sick 0 – 2 months children requiring referral, adopted from CBNC guidelines for Ethiopia [21, 22].

**Additional file 2: Supplementary table 2. **Childhood conditions requiring urgent referral for children 2–59 months (Adopted from the revised IMNCI guidelines for the Ethiopia) [21, 22].

## Data Availability

Data from this study are owned by Hawassa University and stored in a depository at the University. Data can be accessed from the University upon reasonable request.

## Abbreviations

CBNC: Community-Based Newborn Care
CHW: Community Health Workers
FGD: Focus Group Discussion
FMOH: Federal Ministry of Health of Ethiopia
HEP: Health extension program
HEW: Health extension worker
ICCM: Integrated community case management
IDI: In-depth interview
IMNCI: Integrated management of newborn and childhood illness
PSBI: Possible serious bacterial infection
SDG: Sustainable development goal
SNNPR: Southern, Nations, Nationalities and Peoples Region
SYIs: Sick young infants
VSD: Very severe disease
WHO: World Health Organization

## References

[1] United Nations Inter-agency Group for Child Mortality Estimation (UNIGME). ‘Levels & trends in child mortality: report 2019, estimates developed by the United Nations inter-agency group for child mortality estimation.’ New York: United Nations Children’s Fund; 2019.

[2] Ruducha J, Mann C, Singh NS, Gemebo TD, Tessema NS, Baschieri A (2015). Articles How Ethiopia achieved Millennium Development Goal 4 through multisectoral interventions: a Countdown to 2015 case study. Lancet Glob Heal.

[3] Ethiopian Public Health Institute (EPHI) [Ethiopia] and ICF. Ethiopia Mini Demographic and Health Survey 2019: Key Indicators. Rockville, Maryland, USA: EPHI and ICF. 2019. Available from: https://www.dhsprogram.com/pubs/pdf/PR120/PR120.pdf. Accessed on 24 Dec 2020.

[4] Deribew A, Tessema GA, Deribe K, Melaku YA, Lakew Y, Amare AT, Abera SF, Mohammed M, Hiruye A, Teklay E, Misganaw A, Kassebaum N (2016). Trends, causes, and risk factors of mortality among children under 5 in Ethiopia, 1990–2013: findings from the Global Burden of Disease Study 2013. Popul Health Metr.

[5] Lulseged S (2002). Integrated management of childhood illness: a review of the Ethiopian experience and prospects for child health. Ethiop Med J.

[6] Marsh D, Nefdt R, Hazel E (2014). Medical special issue: integrated community case management (iCCM) at scale in Ethiopia: evidence and experience. Ethiop Med J.

[7] Mathewos B, Owen H, Sitrin D (2017). Community-Based interventions for newborns in Ethiopia (COMBINE): cost-effectiveness analysis. Health Policy Plan.

[8] Cervantes K, Salgado R, Choi M, Kalter HD. Rapid Assessment of Referral Care Systems: A Guide for Program Managers. Published by the Basic Support for Institutionalizing Child Survival Project (BASICS II) for the United States Agency for International Development. Arlington; 2003. Accessed 11 Oct 2020.

[9] Amouzou A, Hazel E, Shaw B, Miller NP, Tafesse M, Mekonnen Y (2016). Effects of the integrated Community Case Management of Childhood Illness Strategy on Child Mortality in Ethiopia: A Cluster Randomized Trial. Am J Trop Med Hyg.

[10] Lal S, Ndyomugenyi R, Paintain L, Alexander ND, Hansen KS, Magnussen P, Clarke SE. Community health workers adherence to referral guidelines: evidence from studies introducing RDTs in two malaria transmission settings in Uganda. Malaria J. 2016;15:1–13. 10.1186/s12936-016-1609-7 PMC5121932 27881136

[11] Gilroy KE, Callaghan-koru JA, Cardemil CV, Nsona H, Amouzou A, Mtimuni A, Bryce J. Quality of sick child care delivered by Health Surveillance Assistants in Malawi. Health Policy Plan. 2013;28:573–85. 10.1093/heapol/czs095 PMC3753880 23065598

[12] Owais A, Sultana S, Stein AD, Bashir NH, Awaldad R, Zaidi AKM. Why do families of sick newborns accept hospital care ? A community-based cohort study in Karachi, Pakistan. J Perinatol. 2011;31:586–92. 10.1038/jp.2010.191 PMC3152606 21273989

[13] Hailegebriel TD, Mulligan B, Cousens S (2017). Effect on neonatal mortality of newborn infection management at health posts when referral is not possible. Glob Heal Sci Pract.

[14] English L, Miller JS, Mbusa R, Matte M, Kenney J, Bwambale S (2016). Monitoring iCCM referral systems: Bugoye Integrated Community Case Management Initiative (BIMI) in Uganda. Malar J.

[15] Ilboudo TP, Chou Y, Huang N (2012). Assessment of providers ’ referral decisions in Rural Burkina Faso: a retrospective analysis of medical records. BMC Health Serv Res.

[16] Strachan CE, Nuwa A, Muhangi D, Okui AP, Helinski MEH, Tibenderana JK (2018). Community understanding of the concept of pre-referral treatment and how this impacts on referral related decision-making following the provision of rectal artesunate: a qualitative study in western Uganda. BMC Health Serv Res.

[17] Uwemedimo OT, Arpadi SM, Chhagan MK, Kauchali S, Craib MH, Bah F (2014). Compliance with referrals for non-acute child health conditions: evidence from the longitudinal ASENZE study in KwaZulu Natal, South Africa. BMC Health Serv Res.

[18] Nanyonjo A, Bagorogoza B, Kasteng F, Ayebale G, Makumbi F, Tomson G (2015). Estimating the cost of referral and willingness to pay for referral to higher-level health facilities: a case series study from an integrated community case management programme in Uganda. BMC Health Serv Res.

[19] Newbrander W, Ickx P, Werner R, Mujadidi F (2012). Compliance with referral of sick children: a survey in five districts of Afghanistan. BMC Pediatr.

[20] Nalwadda CK, Guwatudde D, Waiswa P, Kiguli J, Namazzi G (2013). Community health workers – a resource for identification and referral of sick newborns in rural Uganda. Trop Med Int Heal.

[21] Creswell J, Plano Clark V (2011). Designing and conducting mixed methods research.

[22] Federal Ministry of Health, Ethiopia (FMOH). Integrated Community Case Management Training for Health Extension Workers Facilitators Guide. 2018.

[23] FMOH. Federal Ministry of Health, Ethiopia. Integrated Management of Newborn and Childhood Illness, Part 1. 2011. Available from: https://www.open.edu/openlearncreate/-pluginfile.php/71990/mod_resource/content/2/IMNCI_Part_1_Final_Printready_March_2011_.pdf. Accessed 15 Aug 2020.

[24] Beyene H, Hailu D, Tadele H, Persson L, Berhanu D (2020). Insufficient referral practices of sick children in Ethiopia based on a cross-sectional survey. Acta Paediatr.

[25] Southern Nations. Nationalities and Peoples Regional Health Bureau, Ethiopia. Annual report of 2011 E.C./2018-19 G.C.

[26] World Health Organization (WHO). Improving pediatric referral care. WHO Bull. 2008. Retrieved from: WHO | Improving pediatric referral care in the context of child survival activities and IMCI.

[27] Ritchie J, Lewis J, Elam, Ritchie J, Lewis J (2003). Gillian. Designing and selecting samples. Qualitative research practice. A guide for social science students and researchers.

[28] Mason M Sample Size and Saturation in PhD Studies Using Qualitative Interviews _ Mason _Forum_ Qualitative Social Research. 2010.

[29] Graneheim UH, Lundman B (2004). Qualitative content analysis in nursing research: concepts, procedures and measures to achieve trustworthiness. Nurse Educ Today.

[30] Druetz T, Siekmans K, Goossens S. The community case management of pneumonia in Africa: a review of the evidence. Published by Oxford University Press in association with The London School of Hygiene and Tropical Medicine. Health Policy Plan. 2015;30:253–66. 10.1093/heapol/czt104 PMC4325533 24371218

[31] Walter ND, Lyimo T, Skarbinski J, Metta E, Kahigwa E, Flannery B, Dowell SF, Salim AS, Kachurc P (2009). Why first-level health workers fail to follow guidelines for managing severe disease in children in the Coast Region, the United Republic of Tanzania. Bull World Health Organ.

[32] Teklu AM, Litch JA, Tesfahun A, Wolka E, Tuamay BD, Gidey H (2020). Referral systems for preterm, low birth weight, and sick newborns in Ethiopia : a qualitative assessment. BMC Pediatr.

[33] Nair M, Yoshida S, Lambrechts T, Boschi-pinto C, Bose K, Mason EM, Mathai M. Facilitators and barriers to quality of care in maternal, newborn and child health: a global situational analysis through metare-view. BMJ Open. 20014;4:e004749. 10.1136/bmjopen-2013-004749. 10.1136/bmjopen-2013-004749 PMC4039842 24852300

[34] Musa EO, Ejembi CL (2004). Reasons and outcome of paediatric referrals from first- level health facilities in Sabongari, Zaria, Northwestern. J Community Med Prim Heal Care.

[35] United Nations General Assembly (2015). Transforming our world: the 2030 Agenda for Sustainable Development.

[36] Getachew T. Health Extension Workers’ diagnostic accuracy for common childhood illnesses in four regions of Ethiopia: a cross-sectional study. Acta Paediatr. 2019;1–7. 10.1111/apa.14888 PMC7154548 31162734

[37] Guenther T, Mopiwa G, Nsona H, Qazi S, Makuluni R, Fundani CB (2020). Feasibility of implementing the World Health Organization case management guideline for possible serious bacterial infection among young infants in Ntcheu district, Malawi. PLoS ONE.

[38] Kiplagat A, Musto R, Mwizamholya D, Morona D (2014). Factors influencing the implementation of integrated management of childhood illness (IMCI) by healthcare workers at public health centers & dispensaries in Mwanza, Tanzania. BMC Public Health.

[39] Simba DO, Kakoko DC, Warsame M, Premji Z, Gomes MF, Tomson G (2010). Understanding caretakers’ dilemma in deciding whether or not to adhere with referral advice after pre-referral treatment with rectal artesunate. Malar J.

[40] Awasthi S, Kesarwani N, Verma RK, Agarwal GG, Tewari LS, Mishra RK (2020). Identification and management of young infants with possible serious bacterial infection where referral was not feasible in rural Lucknow district of Uttar Pradesh, India: an implementation research. PLoS ONE.

[41] Rahman AE, Herrera S, Rubayet S, Banik G, Hasan R, Ahsan Z (2020). Managing possible serious bacterial infection of young infants where referral is not possible: Lessons from the early implementation experience in Kushtia District learning laboratory, Bangladesh. PLoS ONE.

[42] Accorsi S, Somigliana E, Solomon H, Ademe T, Woldegebriel J, Almaz B (2017). Cost-effectiveness of an ambulance-based referral system for emergency obstetrical and neonatal care in rural Ethiopia. BMC Pregnancy Childbirth.

[43] Applegate JA, Ahmed S, Harrison M, Callaghan-Koru J, Mousumi M, Begum N (2020). Caregiver acceptability of the guidelines for managing young infants with possible serious bacterial infections (PSBI) in primary care facilities in rural Bangladesh. PLoS ONE.

