# Supplementary material for: A mixed-methods study exploring adherence to the referral of severely sick children in primary health care in Southern Ethiopia
Source: Arch Public Health. 2021 Sep 2;79:159. doi: 10.1186/s13690-021-00681-6 (PMC8414758; doi:10.1186/s13690-021-00681-6)
Supplement: Supplementary file 1 — Additional file 1: Supplementary table 1. CBNC Classification table for sick 0 – 2 months children requiring referral, adopted from CBNC guidelines for Ethiopia [21, 22]. [file 13690_2021_681_MOESM1_ESM.docx]

**Supplementary tables**

Supplementary table 1: CBNC Classification table for sick 0 – 2 months children requiring referral, adopted from CBNC guidelines for Ethiopia (21, 22)

| No. | Signs | Classify as | Treatment Identified  (Actions to be taken by the HEW) |
| --- | --- | --- | --- |
| 1 | - Weight < 1,500gm OR - Gestational age < 32 weeks | Very low birth weight and/or very preterm | - Continue breastfeeding (if not sucking feed expressed breast milk by cup) - Start Kangaroo Mother Care (KMC) - **Refer URGENTLY** with mother to hospital with KMC position |
| 2 | - Not feeding well, OR - History of convulsions/convulsing now, OR - Fast breathing (≥60 breaths per minute), OR - Severe chest in-drawing, OR - Fever (≥37.5°C* or feels hot), OR - Low body temperature (< 35.5°C* or feels cold), OR - Movement only when stimulated or no movement even when stimulated. | Very severe disease/VSD | - Breastfeed more frequently (or expressed breast milk if unable to suck and is conscious) - Advise mother on the need for referral - Give a dose of pre-referral amoxicillin, and gentamycin - **Refer URGENTLY** to hospital/health centre. - Advice mother to cover the baby well, including head with blanket and hold close to her body on the way to hospital - When referral is not possible treat the young infant with amoxicillin and gentamycin for 7 days |
| 3 | - Palms and/or soles yellow, OR - Skin and eyes yellow and baby is < 24 hrs. old, OR - Skin and eyes yellow and baby is ≥14 days old | Severe jaundice | - Breastfeed more frequently - Advise mother on the need for referral - **Refer URGENTLY** to health centre/ hospital - Advice mother to cover the baby well, including head with blanket and hold close to her body on the way to hospital /health centre |
| 4 | Two of the following signs:   - - - - Movement only when stimulated, or no movement even when stimulated       - Sunken eyes       - Skin pinch goes back very slowly | Severe dehydration | - **Refer URGENTLY** to health centre/hospital with mother giving frequent sips of ORS on the way. - Advise mother to breastfeed more frequently & longer - Advise mother to keep young infant warm |

Supplementary table 2: Childhood conditions requiring urgent referral for children 2–59 months (Adopted from the revised IMNCI guidelines for the Ethiopia) (21, 22).

| S.N | SIGNS | Classified as | Treatment Identified  (Actions to be taken by the HEW) |
| --- | --- | --- | --- |
| 1 | Any general danger sign  *(Convulsion, Not able to eat or drink, Lethargic, Vomits everything)* | Very severe disease | - Give diazepam if convulsing now - Quickly complete the assessment - Give appropriate pre-referral treatment immediately - Treat to prevent low blood sugar - Keep the child warm - **Refer URGENTLY**. |
| 2 | Any general danger sign OR  • Stridor in calm child | Severe pneumonia or very severe disease | - Give first dose of IV/IM Ampicillin and gentamycin* - **Refer URGENTLY** to hospital** |
| 3 | Two of the following signs:  • Lethargic or unconscious  • Sunken eyes  • Not able to drink or drinking poorly  • Skin pinch goes back very slowly | Severe dehydration | If child has no other severe classification: -   - Give fluid for severe dehydration (Plan C).   OR   - If child also has another severe classification: - **Refer URGENTLY** to hospital with mother giving frequent sips of ORS on the way. - Advise the mother to continue breastfeeding. ► If child is 2 years or older, and there is cholera in your area, give antibiotic for cholera |
| 4 | Dehydration present | Severe persistent diarrhea | - Treat dehydration before referral unless the child has another severe classification - Give Vitamin A - **Refer to hospital** |
| 5 | Any general danger sign, OR  • Stiff neck, OR  • Bulging fontanels (< 1 yr.) | Very severe febrile disease | - Give first dose Artesunate or Quinine for severe malaria - Give first dose of IV/IM Ampicillin and Gentamycin - Treat the child to prevent low blood sugar - Give Paracetamol in health facility for high fever (≥38.5°C) - **Refer URGENTLY** to hospital |
| 6 | Any general danger sign, OR  Clouding of cornea, or  Deep or extensive mouth ulcers | Severe complicated measles ****  *(****other important complication of measles-pneumonia, stridor, diarrhea, ear infection, and acute)* | - Give Vitamin A, first dose - Give first dose of IV/IM Ampicillin and Gentamycin - If clouding of the cornea or pus draining from the eye, apply Tetracycline eye ointment - **Refer URGENTLY** to hospital |
| 7 | Tender swelling behind the ear | Mastoiditis | - Give first dose of Ampicillin and Chloramphenicol IV/IM - Give first dose of Paracetamol for pain - **Refer URGENTLY** to hospital |
| 8 | Severe palmar pallor | Severe anemia | - **Refer URGENTLY** to hospital |
| **9** | **IN INFANTS < 6 MONTHS**   - WFL <-3Z score, and   presence of complications  OR   - Edema of both feet | Complicated severe acute malnutrition | - Give first dose of Ampicillin and Gentamycin IM - Treat the child to prevent Low Blood Sugar - Advise mother on the need of referral - **Refer Urgently** to Hospital |
| **10** | **IN CHILDREN 6 - 59 MONTHS**   - WFL/H < -3Z score or MUAC <11 cm or edema of both feet (+, ++), and any of the following:   • Any one of the medical complications , or  • Failed Appetite test   - +++ edema OR - Marasmic-Kwashiorkor (WFL/H < -3Z with edema or MUAC<11 cm with edema) | Complicated severe acute malnutrition | - Give 1st dose of Ampicillin and Gentamycin IM - Treat the child to prevent low blood sugar - Advise the mother to feed and keep the child warm - Advise mother on the need of referral - **Refer Urgently** to Hospital or admit to inpatient care |
